# Beyond scents: calling on the fragrance industry to champion plant diversity

**DOI:** 10.1093/biosci/biag014

**Published:** 2026-03-20

**Authors:** Luiza F A de Paula, Rhian J Smith, Vanessa Handley, Tiago P Gomes, Raphael Ocelli Pinheiro, Alexandre Antonelli, Peggy L Fiedler

**Affiliations:** Department of Genetics, Ecology and Evolution, Federal University of Minas Gerais, 6627 Pres. Antônio Carlos Avenue, Pampulha, Belo Horizonte, MG, 31270-901, Brazil; The Red List Project, 870 Creed Road, Oakland, CA, 94610, USA; Royal Botanic Gardens, Kew, Richmond, Surrey TW9 3AE, UK; The Red List Project, 870 Creed Road, Oakland, CA, 94610, USA; Montgomery Botanical Center, 11901 Old Cutler Road, Coral Gables, FL, 33156, USA; Fama re.capital, 134 Olimpíadas Street, conj. 42, Vila Olímpia, São Paulo, SP, 04551-000, Brazil; Department of Ecology, Federal University of Rio Grande do Norte, Campus Universitário Lagoa Nova, Rua das Biociências, Natal, RN, 59078-970, Brazil; Royal Botanic Gardens, Kew, Richmond, Surrey TW9 3AE, UK; Gothenburg Global Biodiversity Centre, Department of Biological and Environmental Sciences, University of Gothenburg, Box 463, 405 30, Gothenburg, Sweden; Department of Biology, University of Oxford, South Parks Road, Oxford, OX1 3RB, UK; The Red List Project, 870 Creed Road, Oakland, CA, 94610, USA

**Keywords:** bioprospecting, corporate engagement, intangible heritage, Kunming–Montreal Global Biodiversity Framework, Red List

## Abstract

Since the Convention on Biological Diversity (1992), international frameworks have emphasized benefit-sharing and private-sector engagement in conservation, a call reinforced by the Kunming–Montreal Global Biodiversity Framework (2022). Yet biodiversity continues to decline, highlighting the need for funding models that effectively link commerce with conservation. Here, we examine how the fragrance industry—a sector deeply dependent on plant diversity and cultural value chains—can contribute more meaningfully to global plant conservation. Drawing on international policy frameworks, published literature, and illustrative conservation–industry partnerships, we assess mechanisms through which fragrance-related initiatives support biodiversity protection, accountability, and culturally grounded conservation narratives. We then examine The Red List Project as a conservation-first model that integrates biodiversity objectives directly into fragranced product development, using scent inspiration rather than wild harvesting. We argue that scaling such approaches could reposition the fragrance industry as an active partner in safeguarding plant diversity, biocultural heritage, and equitable benefit-sharing.

The Convention on Biological Diversity (CBD) under the United Nations Environment Programme (UNEP 1992), adopted at the 1992 Earth Summit, established global goals for biodiversity conservation, sustainable use, and equitable benefit sharing, later expanded by the Cartagena Protocol on Biosafety (UNEP 2000) and the Nagoya Protocol on Access and Benefit Sharing (SCB 2011). The International Union for the Conservation of Nature (IUCN) and the Intergovernmental Science-Policy Platform on Biodiversity and Ecosystem Services (IPBES 2019) further strengthened global conservation assessments, incorporating Indigenous and local knowledge. Most recently, the 2022 Kunming–Montreal Global Biodiversity Framework (GBF; CBD 2022) provided a comprehensive strategy to reverse biodiversity loss, setting four global goals for 2050 and 23 individual targets for 2030.

Two GBF targets are particularly relevant to engaging for-profit businesses: Target 15 (accountability and transparency of corporate biodiversity impacts, including benefit-sharing compliance) and Target 19(c) (substantially increasing biodiversity finance through private and blended funding). These targets signal that businesses—especially those reliant on biodiversity—must go beyond risk mitigation to become active contributors to conservation.

Despite these frameworks, biodiversity loss continues at an accelerating pace (Díaz et al. 2019, Ceballos et al. 2020, Cowie et al. 2022, Williams et al. 2022), and conservation funding continues to be profoundly inequitable across taxa (Guénard et al. 2025). It is estimated that *c*. 45% (*c*. 150,000 species) of the world’s flowering plants are at risk of extinction (Bachman et al. 2024), whereas three out of four undescribed plant species are also at risk of extinction (Brown et al. 2023). Although conservation efforts yield some positive, local outcomes—such as invasive species control, habitat protection, and restoration (Langhammer et al. 2024)—global interventions remain insufficient to address biodiversity loss. Clearly, accelerated action and innovative approaches are urgently needed to fully realize the goals of the GBF.

A promising avenue is the development of new tools for resource allocation and accountability. For instance, Hughes and colleagues (2025) propose a globally centralized database of all conservation funding, designed with universal standards and guidelines to prioritize threatened species. In addition, one example of funding innovation is the Cali Fund, a landmark agreement reached at COP16 in Colombia in 2024. The Fund encourages (rather than obliges) large companies using digital sequence information (DSI) from biodiversity—for example, in the pharmaceutical, cosmetics, and nutraceutical industries—to contribute 1% of their profits or 0.1% of their revenue to a UN-hosted trust fund. The Cali Fund is dedicated to fair and equitable benefit-sharing from the use of DSI, with a portion earmarked for Indigenous Peoples and local communities to support conservation, capacity-building, stewardship, and other biodiversity-related initiatives (CBD 2024).

This growing corporate engagement, alongside persistent biodiversity decline, highlights both an urgent need and a compelling opportunity: industries that depend on biodiversity must become genuine partners in conservation. Essential oils and plant-derived fragrance ingredients illustrate this potential vividly. Roughly 2000 plant species supply essential oils for the manufacturing of fragrance accords (the building blocks of perfumes) (Alpha Aromatics 2019), and estimates of plant-derived fragrant molecules exceed 3000 (Lim et al. 2018, Kliszcz et al. 2021). Beyond chemistry, fragrances embody cultural meanings, connecting landscapes, practices, traditions, and sensory experiences, representing both a cultural ecosystem service (Daniel et al. 2012, Oleszkiewicz et al. 2021, King et al. 2025) and a form of intangible heritage (Jung 2015, Bembibre and Strlič 2017). Yet olfactory heritage has historically been both neglected and undervalued compared with visual or auditory dimensions, despite evidence of structured efforts to document and safeguard it (Verbeek 2021; Herz and Bajec 2022).

The fragrance sector’s deep reliance on botanical diversity and cultural value chains positions it as a powerful, yet underexploited, partner for advancing biodiversity goals. We examine how this industry can move beyond narrow supply-chain sustainability concerns and embed conservation finance, accountability, and benefit-sharing into its practices. The fragrance sector could help reshape the relationship between commerce and biocultural diversity stewardship—transforming conservation from a peripheral concern into an integral element of global fragrance production and commercial use.

## Bioprospecting and biopiracy

Humans have interacted with and exploited plants over millennia (Levis et al. 2017), relying on them for an extraordinary diversity of purposes, from food and medicine to materials and social uses (Pironon et al. 2024). Likewise, the commercial development of plant-derived products has yielded countless pharmaceuticals, perfumes, cosmetics, agrochemicals, and functional foods. Although the human benefits of many of these products are undeniable, the exploration of biodiversity for commercialization, also termed bioprospecting, has rightly fallen under increasing scrutiny (Neimark 2017).

During the 1992 Earth Summit, bioprospecting gained new prominence and negative attention. The historical exploitation of biodiversity by high-income countries and unethical practices in the corporate sector prompted some nations to restrict or even prohibit bioprospecting activities (Moran et al. 2001, Neimark and Tilghman 2014). This complex history and the associated inequities are both well-known and irrefutable (e.g., Tripathi and Pandey 2017). The term “biopiracy” was coined to describe the situation where knowledge and biological resources of Indigenous Peoples and other traditional and local communities are appropriated for exclusive control by outside individuals or institutions. Beyond denouncing such clear cases of exploitation, the term biopiracy has also come to be applied much more broadly, as a critique of many forms of legitimate research and commercialization of plants (and, to a lesser degree, animals) (Neimark 2017). In this context, researchers and companies are increasingly forced to anticipate and address potential allegations by adopting stronger ethical standards and legal compliance measures.

For example, Bourdy and colleagues (2017) provide a thorough overview of the *Quassia amara* L. (Simaroubaceae) biopiracy allegation, particularly with respect to patent claims for a bioactive compound that has shown potential in treating malaria and cancer. The study addresses the ethical, legal, and conservation implications of commercializing Indigenous knowledge and genetic resources, highlighting the ongoing debate on bioprospecting versus biopiracy. The discovery and commercialization of the broad-ranging therapeutic compounds of the rosy periwinkle (*Catharanthus roseus* (L.) G.Don; Apocynaceae) is another well-known case study. The ongoing debate over fair distribution of royalties remains politically and scientifically contentious, with conflicting claims and counterclaims regarding benefit-sharing (Neimark 2017). These debates are further complicated in cases where the original plant source is native to one country, but commercialization is initiated elsewhere through cultivated material (e.g., from an *ex situ* collection), raising unresolved questions about who should rightfully receive compensation.

Many seminal papers (e.g., McAfee 1999, McCauley 2006, 2012) cautioned against assigning monetary value to nature and its services, describing the trade of local biodiversity on international markets as “selling nature to save nature.” Although the neoliberal, international commodification of natural capital remains contested and significant capital investment is virtually nonexistent (Dempsey and Suarez 2016), a balanced approach to bioprospecting, acknowledging both its risks and its potential, appears more likely to inspire the conservation actions called for by the CBD and the GBF. In today’s world, transformative solutions are urgently needed, underpinned by compelling storytelling. Blanket criticisms of monetization risks may well be stifling responsible efforts to harness private capital or to meet several GBF targets (Dempsey and Suarez 2016), such as sustainable wild harvesting (Target 5), sustainable management and use of wild species (Target 9), and the integration of biodiversity values into business and financial policies (Target 15). Scaling up positive incentives for biodiversity conservation while simultaneously reforming harmful ones (Target 18) requires a more nuanced and constructive approach.

In this context, the fragrance sector emerges as a particularly relevant partner to reimagine how plant-based industries can move beyond extractive paradigms. With its reliance on thousands of aromatic species and its deep entanglement with cultural heritage, the sector has both the responsibility and the opportunity to pioneer models of equitable collaboration. Fragrance production can demonstrate how commercial success and conservation stewardship are not mutually exclusive but mutually reinforcing if benefit-sharing, accountability, and conservation finance are treated as integral to business practice. This shift lays the foundation for exploring how industry-wide standards, transparency, and innovative funding mechanisms might transform this business sector into a driver of biocultural diversity protection.

## From metrics to cultural value chains

For-profit industries that rely heavily on plants are central to achieving the GBF’s vision that “Nature can be conserved, restored, and used sustainably while other global societal goals are simultaneously met through urgent and concerted efforts fostering transformative change” (Kunming-Montreal Global Biodiversity Framework, Section A.2, paragraph 4, 2022). The need for businesses to become part of the solution to the biodiversity crisis is repeatedly underscored in the GBF (e.g., Target 15) and was emphatically reaffirmed at COP16 in late 2024. To move beyond superficial sustainability claims, companies must embed biodiversity conservation in their core strategies, rather than treating it as a peripheral environment, social, and governance (ESG) item.

Although traditional ESG metrics—emissions reduction, waste minimization, or supply-chain audits—are necessary, they remain insufficient without concrete actions that actively restore and protect ecosystems and the communities dependent on them (UEBT 2023). Effective corporate biodiversity reporting should prioritize measurable outcomes and be supported by standardized guidelines and robust regulatory frameworks (Mair et al. 2024). Without such consistency, reporting remains uneven and fragmented, and investors lack the reliable data needed to make decisions (Adler et al. 2017, Krueger et al. 2023). Importantly, accountability and transparency form the foundation for mobilizing credible private-sector financing: without reliable information on biodiversity impacts, partnerships risk being superficial and ineffective.

Frameworks such as the Dasgupta Review (2021), the Taskforce on Nature-Related Financial Disclosures (TNFD 2023), and the Natural Capital Protocol (Whitaker 2018) now provide guidance for integrating biodiversity into financial systems. Mechanisms like biodiversity credits and payments for ecosystem services (PES) illustrate how incentives can be institutionalized, as seen in Costa Rica’s long-standing PES program (Pagiola 2008) or the system Biodiversity Credits Australia (2023). Yet persistent challenges remain: high-integrity biodiversity markets require transparent reporting and repeatable metrics to avoid the pitfalls that have undermined carbon markets (Peacock 2023, Antonelli et al. 2024).

Again in this context, the fragrance industry provides a compelling case. Its direct dependence on many plant species, combined with its cultural significance, offers both responsibility and opportunity. Several beauty sector businesses, including charitable foundations from major brands (e.g., Aēsop Foundation and Fondation L'Oréal), as well as many smaller companies, have already begun to engage in conservation partnerships (table 1). For example, Caswell-Massey, International Flavors & Fragrance (IFF), and Yellowstone National Park launched the “Yellowstone Living Florals Collection,” which raised funds over 5 years (2019–2023) for the Yellowstone Forever Institute (J. Jarvis personal communication). At a broader scale, IFF and Reservas Votorantim (RV)—the green-economy company within the Votorantim portfolio—have recently established an unprecedented research and bioprospecting partnership at Legado das Águas, a private reserve in the Brazilian Atlantic Forest, managed by RV. The agreement grants IFF exclusive access to the reserve’s flora in exchange for funding an on-site research laboratory dedicated to sustainable ingredient innovation (IFF 2025). With the fragrance market projected to grow from $60.73 billion in 2025 to $101.47 billion by 2034 (Precedence Research 2025), the industry has enormous potential to scale awareness and channel resources into plant conservation.

**Table 1.** Examples of recent existing partnerships linking the fragrance industry with plant conservation and cultural heritage.

Initiatives/country	Actors (institutions, companies, foundations, individuals)	Mechanism	Conservation outcome	Potential and/or contextual cultural value
The Yellowstone Living Florals collection/USA^a^	Caswell-Massey, International Flavors & Fragrances (IFF), Yellowstone Forever	Product-linked fundraising (portion of fragrance sales reinvested in park conservation)	Funding directed to habitat restoration and species protection in Yellowstone National Park	Fragrance collection reinforces the Greater Yellowstone Ecosystem as a cultural and natural heritage icon
Bioprospection in Legado das Águas Reserve/Brazil^b^	International Flavors & Fragrances (IFF), Reservas Votorantim	IFF’s exclusive access to native flora for the development of new extracts; construction of on-site research laboratory	Research and innovation for sustainable fragrance ingredients; conservation of Atlantic Forest biodiversity through controlled access	Ecotourism potential; reinforces value of Brazilian biodiversity as a cultural and economic asset
Musée International de la Parfumerie/France^c^	Musée International de la Parfumerie (Grasse)	Exhibitions, collections, and cultural programming	Preservation of knowledge on plant resources used in perfumery	The Museum takes an anthropological approach to fragrance history, covering raw materials, manufacturing, industry, innovation, trade, design, and uses, showcased through diverse cultural artifacts
“100 Fragrant Sceneries”/Japan^d^	Japan’s Environment Ministry	Heritage branding (designation of culturally significant fragrance landscapes)	Raises awareness of plant species and ecosystems underlying iconic scents	Reinforces cultural legitimacy of fragrance linked to landscapes; strengthens place-based identity
“Original Thüringer Rosenwasser” (Thuringian rose water)/Germany^e^	Rosenhof Holzhausen, UNESCO Global Geopark Thuringia Inselsberg–Drei Gleichen	Heritage branding, geopark-linked certification	Supports cultivation of traditional *Rosa* species; sustains local farming practices	Reinforces cultural legitimacy of fragrance linked to landscapes; connects regional product to UNESCO recognition
(1) Pura x Dutjahn collection; (2) Youth Ranger Program/Australia^f^	(1) K Farmer Dutjahn Foundation, Pura; (2) K Farmer Dutjahn Foundation, Givaudan Foundation	(1) Annual philanthropic grants and proceeds from a dedicated consumer product line branded as Pura x Dutjahn; (2) Givaudan provides targeted financial support for Martu and Birriliburu youth programs	Advancement of Indigenous solutions to protect and restore native sandalwood stands (*Santalum spicatum*)	(1) Economic support and empowerment of Indigenous Wiluna Martu and Birriliburu Peoples; (2) Youth Ranger Program fosters cultural continuity and environmental stewardship among Indigenous youth
Aēsop Foundation/Global^g^	Aēsop Foundation, with 24 different partners, including five environmental groups	Philanthropic grants supporting biodiversity and community initiatives	Funding for youth education, human rights, and environmental conservation with an emphasis on climate change adaptation	For environmental projects, promotion of cultural narratives through support of community-led storytelling and heritage projects
For Women in Science/Global^h^	Fondation L’Oréal; UN Educational, Scientific, and Cultural Organization (UNESCO)	Corporate grants exclusively for women in STEM fields, including environmental science	Support for conservation research, restoration initiatives, and biodiversity awareness campaigns	Empowerment of women scientists and cultural recognition of biodiversity-linked knowledge
“Resurrecting the Sublime”/Global^i^	Daisy Ginsberg (architect and designer), Sissel Tolaas (scent researcher and artist), Ginkgo Bioworks (biotech company)	Synthetic biology and art installation: recreating scents of extinct flowers and exhibiting them in museums/galleries	Raises awareness of extinct and threatened plant lineages; stimulates interest in conservation of related taxa	Restores sensory memory of lost species; frames extinct flora as cultural heritage and prompts reflection on ecological loss

*Note:* The table illustrates diverse mechanisms—from product-linked fundraising and bioprospecting agreements to museums, heritage branding, and philanthropic foundations—that demonstrate how the fragrance sector and related institutions can contribute to biodiversity protection while reinforcing cultural narratives and intangible heritage.

a International Flavors & Fragrances (IFF). *Partnership with Caswell-Massey* and *Yellowstone Forever*. Available at: https://iff.gcs-web.com/news-releases/news-release-details/caswell-massey-iff-yellowstone-forever-launch-yellowstone-living (accessed 26 January 2026).

b International Flavors & Fragrances (IFF). *Partnership with Reservas Votorantim*. Company press release. Available at: https://www.iff.com/media/news/iff-reservas-votorantim-partnership/ (accessed 26 January 2026).

c Musée International de la Parfumerie. *Collections & Exhibitions*. Available at: https://www.museesdegrasse.com/musee-international-de-la-parfumerie (accessed 26 January 2026).

d Japan Environment Ministry. *100 Fragrant Sceneries Initiative*. Available at: https://www.env.go.jp/en/ (accessed 26 January 2026).

e UNESCO Global Geopark Thuringia Inselsberg–Drei Gleichen. *Original Thüringer Rosenwasser*. Available at: https://geofood.no/geoplaces/thuringia-inselsberg-drei-gleichen/ (accessed 26 January 2026).

f *Pura x Dutjahn collection* and *Youth Ranger Program*. Available at: https://pura.com/blogs/pura/a-vision-to-heal and at https://www.kfdf.com.au/youth-ranger-program (accessed 26 January 2026).

g Aēsop Foundation. Available at: https://www.aesop.com/au/r/aesop-foundation (accessed 26 January 2026).

h Fondation L’Oréal. Available at: https://www.fondationloreal.com (accessed 26 January 2026).

i *Resurrecting the Sublime*. Available at: https://www.scientificamerican.com/article/guerilla-artist-daisy-ginsberg-recreates-scent-of-extinct-flowers/ (accessed 26 January 2026).

Beyond biodiversity-specific mechanisms, financial innovations emerging in other sectors also provide funding models. Sustainability-linked loans, such as Marfrig’s agreement with the Dutch Fund & Green (IDH 2025), tie borrowing conditions directly to conservation targets like traceable, deforestation-free supply chains. Similar approaches could be adapted for fragrance companies to link capital access with measurable biodiversity outcomes.

Equally important is recognizing that plant conservation financing should respect and be informed by cultural legitimacy. Fragrances are not merely commodities; they are embedded in cultural value chains that reflect relationships between people, plants, and places (Xiao 2022). Across the initiatives summarized in table 1, conservation outcomes and cultural values are generated simultaneously through shared mechanisms. Product-linked fundraising initiatives—such as fragrance collections supporting protected areas (e.g., Yellowstone National Park, USA)—translate consumer sales into direct funding for habitat restoration and species protection, while reinforcing iconic landscapes as elements of natural and cultural heritage. Heritage designation programs, including “Japan’s 100 Fragrant Sceneries” and UNESCO-linked regional products (e.g., rose water from the UNESCO Global Geopark Thuringia Inselsberg–Drei Gleichen, Germany), raise awareness of the plant species and ecosystems underlying iconic scents, supporting traditional cultivation practices while strengthening place-based identity and cultural legitimacy. Cultural institutions such as the “Musée International de la Parfumerie” in Grasse, France, contribute to conservation by preserving knowledge of plant resources used in perfumery while simultaneously framing raw materials, trade, and innovation as part of shared cultural history. Cultural outcomes reinforced through processes of institutional legitimation enhance credibility and durability, as they embed fragrance-related conservation narratives within established governance and cultural memory structures, extending their influence beyond market-driven cycles.

A complementary pillar centers on community- and philanthropy-based initiatives—including Indigenous-led partnerships and global foundations (e.g., the “Youth Ranger Program” in Australia, the various biodiversity and community initiatives from Aēsop Foundation, and the For Women in Science program led by Fondation L’Oréal and partners)—that combine financial support for biodiversity protection, education, and restoration with empowerment, cultural agency, and the transmission of biodiversity-linked knowledge (table 1). With due attention to governance arrangements, decision-making processes, and authorship of narratives, these initiatives transform economic visibility into a mechanism for intergenerational continuity, reinforcing the long-term resilience of biocultural diversity systems that rely on sustained stewardship of species and landscapes. Finally, art–science collaborations that seek to reconstruct the scents of extinct flowers, such as the “Resurrecting the Sublime” project (table 1), use sensory experience to raise awareness of plant loss, linking conservation discourse to cultural memory and reflection on ecological change. Together, these examples demonstrate how fragrance-related partnerships can operate beyond formal agreements by coupling tangible conservation action with culturally grounded narratives, allowing cultural outcomes to be assessed as structured and observable effects, and strengthening connections between consumers and landscapes while reinforcing both tangible and intangible values.

Thus, aligning accountability frameworks, biodiversity finance, and cultural heritage positions the fragrance sector as a potential leader in integrating commerce, plant conservation, and cultural stewardship. This integrated approach not only safeguards ecosystems and strengthens supply chains but also prepares the ground for innovative initiatives.

## The Red List Project and the fragrance industry as a case study

The Red List Project (TRLP; www.theredlistproject.org see our disclosure of conflicting interests describing our close associations with TRLP) is a novel model of conservation engagement that exemplifies how the fragrance sector can operationalize accountability, finance, and cultural value chains for biodiversity. TRLP is a US-based nongovernmental organization (NGO) dedicated to the conservation of imperiled plants and critical habitats. Its funding model centers on partnerships with the commercial fragrance sector, compelling this industry to provide direct and genuine support for plant conservation. Collaborations are nucleated around rare and threatened plants or habitats—designations according to the global IUCN Red List and/or national Red Lists in biodiversity hotspots (Myers et al. 2000, Mittermeier et al. 2004)—and their evocative scent profiles. These scents inspire the development of fragrance accords that are then used in commercial products that generate proceeds for conservation actions for the focal plant or site, whereas the accompanying storytelling raises awareness of each species’ ecological and cultural significance (table 2).

**Table 2.** Current examples of fragrance-supported plant conservation projects of The Red List Project (TRLP) across global biodiversity hotspots.

Country/biodiversity hotspot	Species/focus	Conservation status	Industry partner	Local/academic partner	Product developed	Conservation action/outcome	Potential and/or contextual cultural value
Brazil/Atlantic Forest^a^	*Alstroemeria caryophyllaea* (Alstroemeriaceae)	Endangered (Brazilian CNCFlora; no global IUCN designation)	Blocki (historic US fragrance manufacturer)	Fundação Antonelli para a Pesquisa e Conservação da Biodiversidade, Federal University of Minas Gerais	Novel fragrance accord development in fine fragrance	Funding for in-country conservation, graduate-level research, and education	Linking of Brazil’s highly threatened Atlantic Forest flora to historic perfumery traditions, elevating a little-known endangered species as a cultural representative for national biodiversity
Ecuador/Chocó Cloud Forests^b^	*Magnolia chiguila, M. mashpi, M. mindoensis* (Magnoliaceae)	*M. chiguila*—IUCN Critically Endangered; *M. mashpi*—IUCN Least Concern; *M. mindoensis*—IUCN Vulnerable	MANE (global fragrance and flavors manufacturer)	Mashpi Reserve & Lodge (Mashpi Cloud Forest Reserve)	Novel fragrance accord currently adopted as an ingredient in bath/body products by several commercial brands	Support for habitat stewardship focusing on development of propagation techniques and establishment of local nurseries, and species conservation programs	Magnolias carry strong symbolic and aesthetic value in Ecuadorian landscapes; storytelling connects reserve ecotourism and local community stewardship with global fragrance markets
Italy/Mediterranean Basin^c^	*Viola ucriana* (Violaceae)	IUCN Critically Endangered	Baruti Perfumes (Boutique European fragrance house)	University of Palermo	Novel fragrance accord developed for ambient spray	Support for development of visitor signage and educational materials for the protected area where the rare plant occurs	Celebrates olfactory heritage of Mediterranean plants, reinforcing the link between traditional uses and modern fragrance innovation
Cuba/Caribbean^d^	*Juniperus barbadensis* subsp. *lucayana* (= *J. barbadensis* var. *lucayana, J. lucayana*) (Cupressaceae)	Critically Endangered (listed by synonym *J. lucayana* in Cuba’s Red List); IUCN Vulnerable	MANE (global fragrance & flavors manufacturer)	Planta! (Environmental NGO)	Novel fragrance accords currently under consideration by various beauty brands	Support for in-country conservation efforts, including native plant nurseries, propagation for reintroduction; genomic research	Draws from rich local traditions of plant use, creating scents that valorize community knowledge and island heritage while raising awareness of global biodiversity hotspots
Jamaica/Caribbean^e^	*Portlandia platantha* (Rubiaceae)	Various, depending upon the country; IUCN Vulnerable	Baruti Perfumes (Boutique European fragrance house)	Natural History Museum of Jamaica -Institute of Jamaica (Department of Botany)	Novel fragrance accord developed for ambient spray	Conservation-linked consumer products support assessment and monitoring work by in-country partner	Draws from rich local traditions of plant use, creating scents that valorize community knowledge and island heritage while raising awareness of global biodiversity hotspots
Guam/Micronesia^f^	*Cycas micronesica* (Cycadaceae)	IUCN Endangered	MANE (global fragrance & flavors manufacturer)	Montgomery Botanical Center	Novel fragrance accords currently under consideration by one or more beauty brands	Field surveys, augmentation of existing *ex situ* collections, and genomic assessment	Draws from rich local traditions of plant use, creating scents that valorize community knowledge and island heritage while raising awareness of global biodiversity hotspots

*Note:* The table summarizes focal species or groups, conservation status, industry and local/academic partners, product developed, conservation actions, and associated potential and/or contextual cultural values. Cultural aspects emphasize how fragrances derived from or inspired by threatened plants can contribute to intangible heritage, local traditions, and global storytelling, reinforcing the role of the fragrance industry in bridging biodiversity conservation with cultural ecosystem services.*Abbreviations:* BGCI, Botanic Gardens Conservation International; Brazilian CNCFLORA, Centro Nacional de Conservação da Flora; IUCN, International Union for Conservation of Nature.

a Additional information for the project in Brazil: Blocki, https://www.blocki.com; Antonelli Foundation for Biodiversity Research & Conservation (Hidden Universe: Biodiversity), www.hu-b.org (accessed 26 January 2026).

b Additional information for the project in Ecuador: MANE, https://www.mane.com/; Mashpi Reserve and Lodge, https://www.mashpilodge.com/the-reserve/ (accessed 26 January 2026).

c Additional information for the project in Italy: Baruti perfumes, https://barutiperfumes.com/ (accessed 26 January 2026).

d Additional information for the project in Cuba: MANE, https://www.mane.com/; Planta!, https://www.planta.ngo/en/ (accessed 26 January 2026).

e Additional information for the project in Jamaica: Baruti perfumes, https://barutiperfumes.com/; Natural History Museum of Jamaica, https://nhmj-ioj.org.jm/ (accessed 26 January 2026).

f Additional information for the project in Guam: MANE, https://www.mane.com/; Montgomery Botanical Center, https://www.montgomerybotanical.org/ (accessed 26 January 2026).

A distinctive feature of TRLP’s approach is its reliance on scent recreation rather than extraction, ensuring novel ingredient creation without wild harvest or collateral ecosystem damage. This partnership model directly advances the GBF’s Target 15 by ensuring that the use, harvesting, and trade of wild species is legal, sustainable, and safe, and in compliance with international conventions and protocols (CBD 2022). In addition, TRLP engages with plant conservation experts from academia, including botanic gardens, natural history museums, and universities, to guarantee that conservation projects are guided by the best available science.

**Figure 1.**
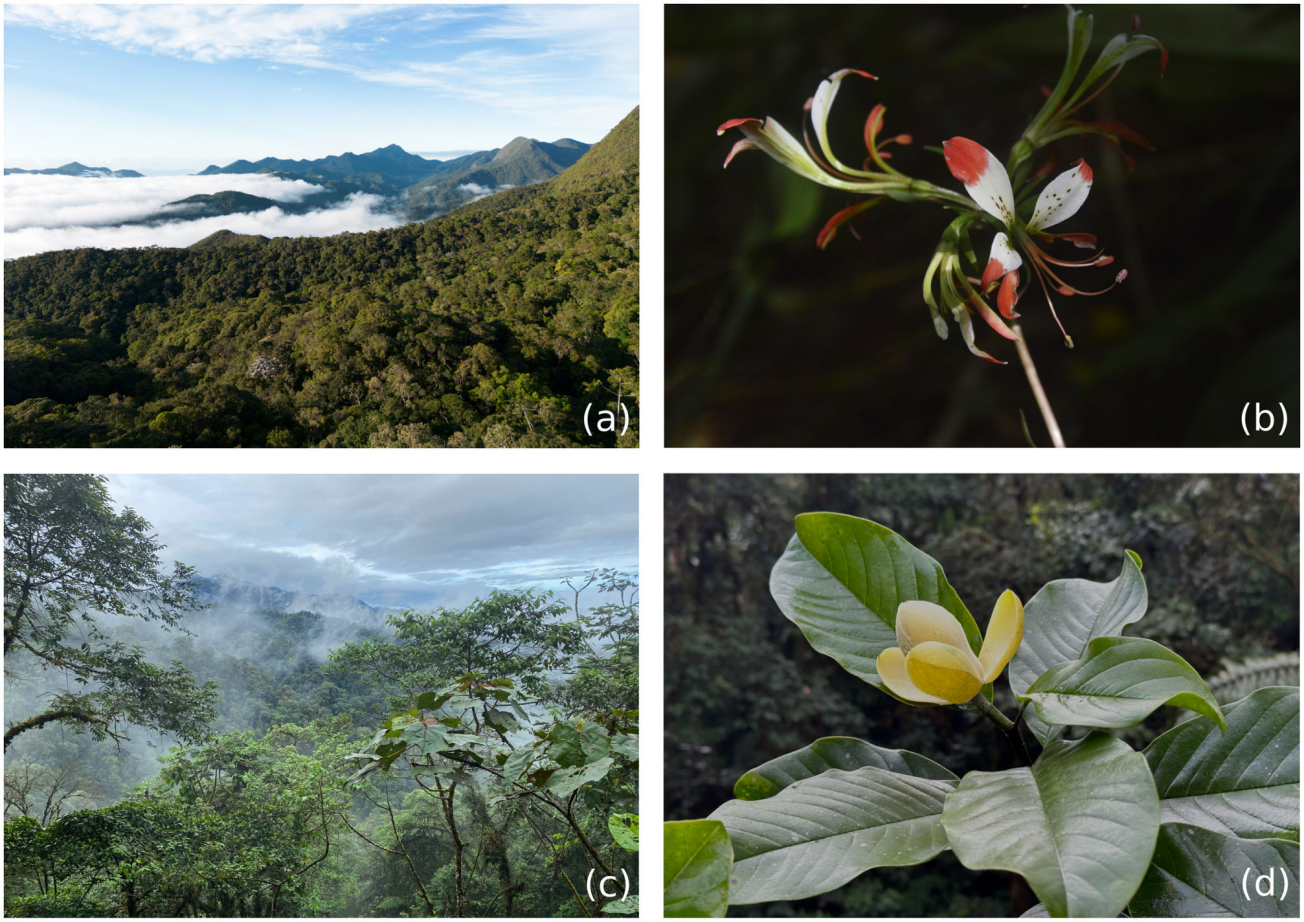
Examples of ecosystems and species studied by The Red List Project in Brazil and Ecuador, respectively. (a) and (b)—The Brazilian Atlantic Forest landscape and the target fragrant *Alstroemeria caryophyllaea* Jacq. (Alstroemeriaceae); (c) and (d)—The tropical montane forest within Masphi Reserve, Ecuador, and the target fragrant *Magnolia mashpi* Á.J.Pérez, F.Arroyo & A.Vázques (Magnoliaceae). Photographs: (a) by A. Antonelli, (b) by L. F. A. de Paula, and (c) and (d) by V. Handley.

Projects have been supported in the Caribbean, Mediterranean Basin, South Pacific, and South America through novel ingredient development and fragranced consumer products (table 2). For instance, in Brazil, TRLP has focused on *Alstroemeria caryophyllaea* Jacq. (Alstroemeriaceae) (figure 1a, 1b), a threatened species from the Atlantic Forest global biodiversity hotspot (Vancine et al. 2024). For this project, TRLP partnered with the historic US fragrance manufacturer Blocki to develop a fine fragrance inspired by the scent of this Brazilian species (table 2). The in-country conservation partners are the Federal University of Minas Gerais (UFMG) and the Fundação Antonelli para a Pesquisa e Conservação da Biodiversidade, part of the “Hidden Universe: Biodiversity” initiative, an NGO dedicated to promoting research, education, conservation, and the sustainable use of biodiversity. Proceeds from this collaboration have supported fieldwork to collect live material for *ex situ* collections, detailed mapping for existing populations, and research conducted for a master’s thesis at UFMG, which is both reassessing the extinction risk of Brazilian *Alstroemeria* species through species distribution modeling and expanding these taxa in *ex situ* collections.

Elsewhere in South America, TRLP has partnered with a global fragrance and flavors manufacturer, MANE, and a local nature reserve, Mashpi Reserve and Lodge, to steward several imperiled *Magnolia* species endemic to the Ecuadorian Chocó (figure 1c, 1d) (table 2). In addition, a previously described collaboration between TRLP, a boutique European fragrance house, and a university scientist exemplifies how such partnerships can directly engage academia in plant conservation (Gianguzzi et al. 2022) (table 2). Such initiatives showcase how industry–academia–NGO collaborations can serve as powerful models for marrying plant conservation with commercial success, fostering both biodiversity protection and sustainable business growth.

## A flexible model for global conservation funding

**Figure 2.**
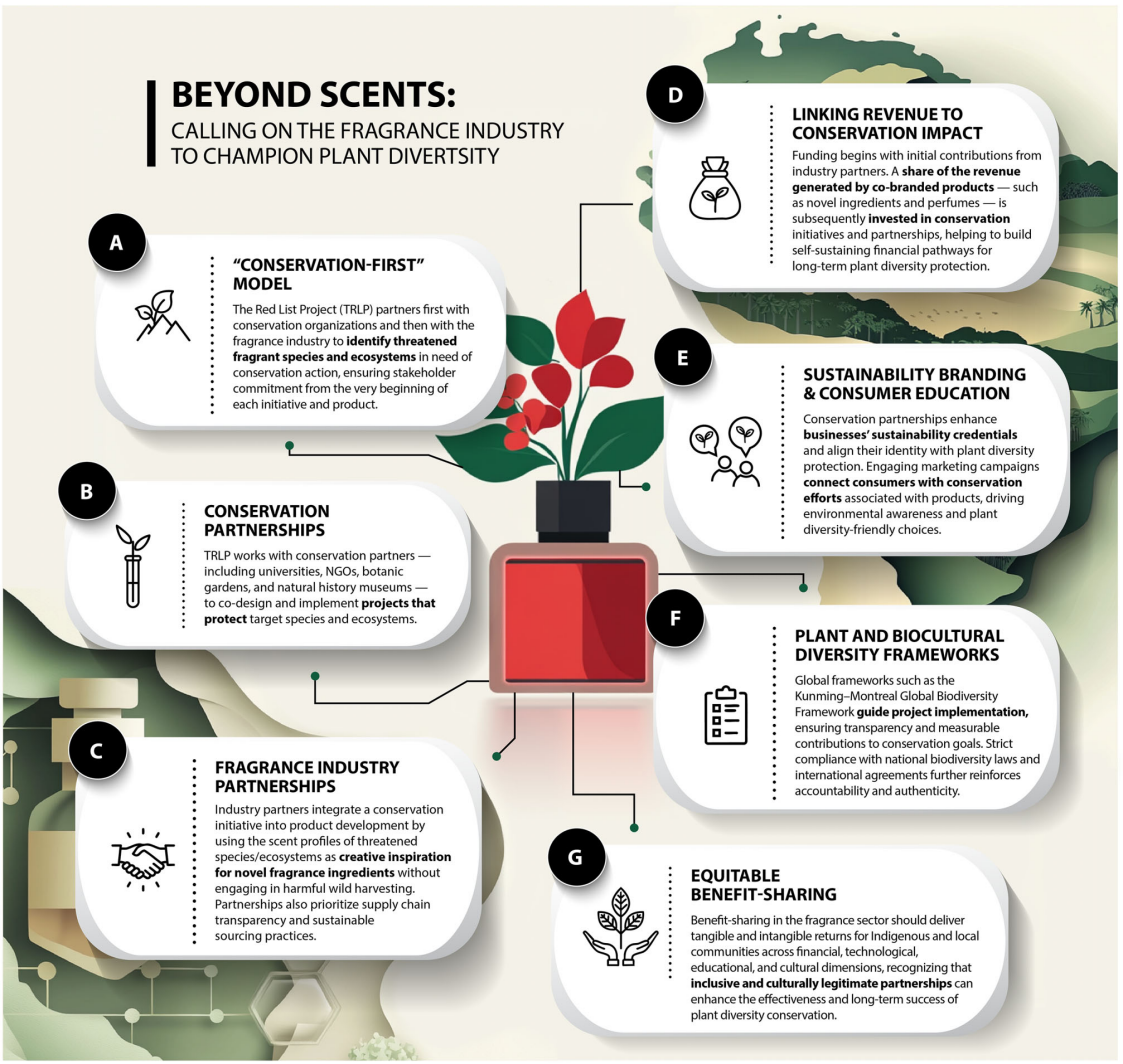
Mechanisms for integrating businesses into biodiversity conservation, illustrated through The Red List Project and the fragrance industry as a case study.

Partnerships like TRLP not only fund plant conservation but also establish replicable models for other industries. Although the impact of such initiatives may appear limited, they act as pilots, testing approaches and providing insights for broader implementation. The cumulative impact of multiple small projects, however, can drive larger-scale action by influencing corporate strategies and government policies to advance conservation efforts effectively. Building on TRLP’s work, we propose several mechanisms (figure 2) that can further enhance corporate contributions to plant conservation, particularly in sectors with plant-dependent supply chains.

Unlike many conservation–industry collaborations that focus primarily on offsetting impacts, TRLP is conceived as a “conservation-first” model (figure 2a), in which partnerships with an in-country conservation organization (figure 2b) and a fragrance industry partner (figure 2c) are established at the very outset to identify threatened fragrant species and ecosystems, ensuring stakeholder commitment from the inception of the initiative (figure 2a). Companies can design fragrance accords (e.g., captured by headspace technology), using the scent profiles of threatened species and ecosystems as inspiration, without resorting to destructive wild harvesting (figure 2c). This approach not only draws value from biodiversity but also ensures that a portion of the revenue is directed back into conservation initiatives co-developed with conservation partners (figure 2b), creating partial or fully self-sustaining funding streams for plant conservation (figure 2d). Conservation is therefore integrated into the entire product lifecycle, guided by sustainability principles at every stage, from concept and sourcing to commercialization and consumer engagement, rather than added later as an optics-driven afterthought.

Essential to TRLP’s model of funding plant conservation projects is an upfront financial contribution from the fragrance industry partner, which TRLP passes through in its entirety to the in-country conservation partner (figure 2d). This requirement to initiate a project ensures mitigating the financial uncertainty that could arise if product sales fluctuate or fail. Proceeds from product sales are provided in regular intervals over the life of the product, and these are also passed through to the conservation entity (figure 2d). The amount of upfront contribution and product sales revenue typically varies with each project.

Additionally, conservation partnerships can serve as powerful branding tools, allowing corporations to genuinely enhance their ESG credentials (figure 2e). TRLP’s collaborations across scales—from boutique fragrance companies to multinational corporations—exemplify how conservation partnerships can align with a company’s identity, bridging commercial goals with tangible biodiversity outcomes. Importantly, consumer education (figure 2e) is an additional critical output in this model, as businesses can engage and inform their consumers about the plant species and ecosystems they are helping to protect, thus driving demand for biodiversity-friendly products. Consumer-facing mechanisms such as eco-labeling, QR-code traceability, biodiversity certification, and awareness campaigns reinforce this connection, linking purchasing choices directly to conservation impact. Furthermore, businesses can leverage existing frameworks such as the GBF, using transparent biodiversity reporting to ensure that their contributions are measurable and impactful (figure 2f). Alongside the GBF, other legal instruments regulating the use of nontimber forest products—such as national biodiversity legislation and international agreements—must also be taken into account (figure 2f).

Although TRLP’s model successfully connects industry to plant conservation, benefit-sharing remains a significant and uneven challenge. Many of the plant species that inspire fragrances are not directly harvested or commercially exploited by local or Indigenous communities, which limits opportunities in specific cases for direct economic return. In such contexts, TRLP’s contributions have focused primarily on capacity building, such as training researchers in the countries of origin and strengthening the expertise of staff in protected areas where these species occur.

Beyond these material dimensions, TRLP initiatives also engage with biocultural narratives, although the nature and evidentiary basis of associated cultural values vary among partnerships. In this context, the cultural values listed in table 2 should be understood as potential or anticipated cultural outcomes, enabling comparisons and analysis across initiatives with differing governance models, geographic contexts, and modus operandi, rather than uniformly documented benefits across all cases. These values are informed by a combination of community-identified priorities (where such information is available), documented experiences from TRLP-affiliated initiatives, and insights from long-term collaborations.

The “Potential/contextual cultural value” column in table 2 reflects this variability. In some partnerships, cultural values are co-defined with local actors and built upon existing symbolic, aesthetic, or landscape-level meanings recognized by communities, as illustrated by *Magnolia* conservation initiatives in Ecuador, where cultural significance is locally articulated and reinforced through stewardship and ecotourism-linked narratives. In other cases, particularly for plant species with no documented traditional use or cultural association, cultural value is interpretive rather than community-derived, articulated primarily by project partners through storytelling that elevates little-known threatened species within national and global conservation imaginaries, like the case of the *Alstroemeria* species in the Brazilian Atlantic Forest. The distinction between these cases underscores the need for more systematic, community-driven evaluation frameworks to ensure that industry–conservation partnerships move beyond optics-driven narratives toward meaningful, reciprocal, and locally accountable forms of benefit-sharing.

Experiences from other sectors, in addition, demonstrate that benefit-sharing can take diverse and innovative forms. In cocoa and coffee value chains, models such as transparent supply chains, cooperative ownership, and direct trading relationships have shown that it is possible to generate more equitable outcomes for producers while maintaining competitive access to global markets (Vicol et al. 2018, Krauss and Barrientos 2021). These examples highlight that benefit-sharing can be embedded into commodity systems and that similar approaches could be adapted by the fragrance industry, which also relies on biodiversity-based raw materials and narratives of authenticity.

Building on these lessons, we propose that benefit-sharing in the fragrance sector should encompass both tangible and intangible returns for Indigenous and local communities across four dimensions (figure 2g). First, financial benefits could include revenue-sharing agreements in which a portion of profits from fragranced product lines inspired by target plant species and ecosystems is reinvested into local conservation funds or livelihood initiatives. In practice, such profits can already be paid into the Cali Fund (COP16 2024), with proceeds directed to Indigenous and local communities. Ownership models that grant farmers or communities equity in companies producing or marketing natural products can also promote fairer value distribution and corporate power (Krauss and Barrientos 2021). Second, technological benefits involve transferring knowledge and equipment for sustainable harvesting or processing, enhancing local control over bioculturally diverse products and fostering community-based conservation (Arvanitidis et al. 2025). Third, educational benefits could be realized through training programs, scholarships, or partnerships that strengthen scientific capacity and empower community members to engage in education, conservation, and monitoring (see Børresen et al. 2023, Valdez et al. 2024). Fourth, cultural benefits might include the recognition and protection of cultural identity and heritages, ensuring that these values are acknowledged in product narratives and, where appropriate, legally safeguarded and actively transmitted to next generations (Ijatuyi et al. 2025).

## Reframing bioprospecting for a sustainable global bioeconomy

Thirty years on from the Earth Summit, with many insights gained along the way, we argue that bioprospecting should be reframed to reflect contemporary ecological, economic, ethical, and social realities. Indeed, there is a need for bioprospecting to move increasingly away from economies that rely heavily on the finite extraction of natural resources, that is, timber, oil, minerals, and agricultural activities that demand large tracts of land. Within emerging policy frameworks—such as those proposed under Brazil’s G20 presidency (G20 2025)—bioeconomies are explicitly framed to prioritize sustainable and inclusive uses of biodiversity, valuing ecosystems not for their extractive potential but for their contribution to long-term environmental resilience and human health and well-being (Jaramillo et al. 2019).

The fragrance industry provides a compelling example at the intersection of science, conservation, for-profit business, and equitable benefit-sharing. Historically reliant on natural raw materials, the sector is increasingly aware of the need for sustainable practices. We advocate for stronger collaboration between governments, industries, and conservation organizations, moving beyond ethical supply chains toward strategies that prioritize biodiversity and center community and Indigenous stakeholders as enduring partners. Following the recommendations from COP16, financial returns from biodiversity-based products, such as fragrances, should be directed toward conservation, restoration, and scientific research, embedding environmental stewardship into the industry’s profit models.

To realize this vision, conservation organizations should also consider adapting by broadening their expertise and deepening engagement with industry partners. We call for more diverse advisory boards and scientific oversight committees that bring together specialists in conservation science, sustainable sourcing, botany, and ecology, alongside representatives of local and Indigenous communities. Equally important is the active involvement of these diverse actors, especially plant scientists, in sectors such as fragrance and beauty, where their knowledge can guide responsible sourcing, prevent overexploitation, and ensure that ecosystems are protected while cultural and economic values are respected. Although supply chain dynamics may seem distant from traditional botanical work, mastering these complexities is critical to safeguarding plant diversity and promoting sustainable bioprospecting.

Plant diversity has supported the evolution and well-being of humans for millennia and will undoubtedly continue to do so for generations to come. Yet without plant diversity underpinning the multitude of essential ecosystem services—from food and medicine to climate regulation and cultural values—human well-being and even societal survival would be fundamentally compromised. Through inclusive and innovative approaches, there is potential for responsible bioprospecting that helps us tackle some of our most pressing societal and environmental challenges, from biodiversity loss to climate change and food security. We urge all stakeholders to move beyond fear and mistrust and embark on an evidence-based, transparent, and mutually beneficial journey to ensure the responsible use of biodiversity while contributing to global conservation goals.

## Author Biography

Luiza F. A. de Paula (luizafap@gmail.com) is a researcher and visiting professor in the Department of Genetics, Ecology, and Evolution at the Federal University of Minas Gerais (Brazil) and a project scientist at The Red List Project (TRLP; USA). Rhian J. Smith is senior science editor and coordinator at the Royal Botanic Gardens, Kew (UK). Vanessa Handley is a biologist at Montgomery Botanical Center and director of conservation science at TRLP (USA). Tiago P. Gomes is a finance professional at Fama re.capital (Brazil). Raphael O. Pinheiro is a researcher and visiting professor in the Department of Ecology at the Federal University of Rio Grande do Norte (Brazil). Alexandre Antonelli is the executive director of science at the Royal Botanic Gardens, Kew (UK), a professor at the Department of Biological and Environmental Sciences, University of Gothenburg (Sweden), a visiting professor at the Department of Biology, University of Oxford (UK), and president of the Antonelli Foundations for Biodiversity Research and Conservation (Brazil). Peggy L. Fiedler is the executive director and principal scientist of TRLP (USA).
